# Repression of insulin gene transcription by indirect genomic signaling via the estrogen receptor in pancreatic beta cells

**DOI:** 10.1007/s11626-019-00328-5

**Published:** 2019-02-21

**Authors:** Takashi Sekido, Shin-ichi Nishio, Yohsuke Ohkubo, Keiko Sekido, Junichiro Kitahara, Takahide Miyamoto, Mitsuhisa Komatsu

**Affiliations:** 1 Division of Diabetes, Endocrinology and Metabolism, Department of Internal Medicine, Shinshu University School of Medicine, 3-1-1 Asahi, Matsumoto, 390-8621 Japan; 2 Miyamoto Clinic, Matsumoto, 390-0841 Japan

**Keywords:** Estrogen receptor, Estradiol, Insulin gene, Repression, Transcription

## Abstract

The mechanism whereby 17β-estradiol (E2) mediates insulin gene transcription has not been fully elucidated. In this study, exposure of hamster insulinoma (HIT-T15) cells to 5 × 10^−9^ to 1 × 10^−7^ M E2 led to a concentration-dependent decrease of insulin mRNA levels. Transient expression of the estrogen receptor (ER) in HIT-T15 cells revealed that estrogen receptor α (ERα) repressed transcription of the rat insulin II promoter in both ligand-dependent and ligand-independent manners. The N-terminal A/B domain of ERα was not required for either activity. However, the repression was absent with mutated ER lacking the DNA-binding domain. Moreover, introducing mutations in the D-box and P-box of the zinc finger of ER (C227S, C202L) also abolished the repression. Deletion of the insulin promoter region revealed that nucleotide positions − 238 to − 144 (relative to the transcriptional start site) were needed for ER repression of the rat insulin II gene. PDX1- and BETA2-binding sites were required for the repression, but an estrogen response element-like sequence or an AP1 site in the promoter was not involved. In conclusion, we found that estrogen repressed insulin mRNA expression in a beta cell line. In addition, the ER suppressed insulin gene transcription in a ligand-independent matter. These observations suggest ER may regulate insulin transcription by indirect genomic signaling.

**Electronic supplementary material:**

The online version of this article (10.1007/s11626-019-00328-5) contains supplementary material, which is available to authorized users.

## Introduction

17-β-Estradiol (E2) evokes diverse biological effects, including the control of development, reproduction, and metabolism, as well as effects on cell growth and differentiation, by acting through the native nuclear receptors estrogen receptor α (ERα), ERβ, and G protein-coupled ER 1 (GPER1), which are present in the cell membrane. ERα and ERβ are members of a large family of nuclear receptors that activate or repress the transcription of hormone-regulated genes upon ligand binding (Evans 1988). Both ERα and ERβ are comprised of a separable N-terminal domain (A and B regions), a DNA-binding domain (DBD, C), a hinge domain (D), a ligand-binding domain (E), and a C-terminal domain (F) (Chambraud et al. 1990). A ligand-dependent activation function in the C-terminal region of the ligand-binding domain (LBD) and a ligand-independent activation function in the N-terminal domain have also been characterized (Tora et al. 1989). The signaling mechanism of estrogen is divided into the following four distinct subtypes (Vrtačnik et al. 2014). (1) Direct genomic signaling. This is a classic estrogen signaling mechanism, where the E2–ER complex binds directly to an estrogen-response element (ERE). In direct genomic signaling, the ER binds as a homodimer to an ERE in the promoter of an estrogen-responsive gene (Kumar and Chambon 1988). Similar to other nuclear receptors, the ER recruits an array of transcriptional cofactors (coactivators and corepressors) that bind the receptor and also interact with other transcription factors, including components of the general transcription factor apparatus (Horwitz et al. 1996). (2) Indirect genomic signaling. The E2–ER complex binds to other transcriptional regulatory factors through protein–protein interactions, and transcription factors bind to their unique responsive elements. Regarding this estrogen-signaling mode, it is known that complexes of JUN, Fos, E2, and ERs activate many genes at the AP1-binding site. Estrogen signals are also transmitted via transcriptional regulatory factors such as Sp1, NFκB, and STAT5 (Bjornstorm and Sjoberg 2005). (3) Non-genomic signaling. E2 binds ERα, ERβ, and GPER1, and the resulting signals are transmitted by the activation of various protein kinase cascades (Heldring et al. 2007). (4) Ligand-independent signaling. E2 is not involved, and transcription of the target gene is initiated by ER phosphorylation and ERE binding (Nilsson et al. 2001).

With indirect genomic signaling, a direct interaction between NFκB and ER has been demonstrated and requires the DBD (C) and D regions of the ER (Stein and Yang 1995). This direct protein binding contributes to interleukin 6 promoter repression by estrogen. Mutational analysis revealed specific residues within the second zinc finger structure of the ER DBD that discriminated between the classical mechanism of ER action and modulation of AP1 and STAT5 activities through tethering (Bjornstorm and Sjoberg 2005).

ER is present in pancreatic β cells (Le May et al. 2006; Alonso-Magdalena et al. 2008; Liu et al. 2009; Martensson et al. 2013). It has been reported that E2 plays important biological roles in pancreatic β cells in mammals (Ropero et al. 2008; Tiano and Mauvais-Jarvis 2012; Liu et al. 2013). E2 increases the pancreatic insulin content through ERα in cultured islets (Alonso-Magdalena et al. 2008). Wong et al. reported that islet ERα enhances insulin biosynthesis in vitro and in vivo, and it amplifies the effect of glucose in stimulating the insulin gene promoter. ERα enhances insulin synthesis in cultured insulinoma (INS-1) cells. It was also demonstrated that non-genomic estrogen signaling contributes to the mechanism of insulin promoter activation. Extranuclear ER stimulates transcription factor BETA2 (Naya et al. 1995) by binding to the insulin promoter and driving insulin synthesis (Wong et al. 2010). It has recently been suggested that short-term exposure of estrogens activates insulin secretion and that long-term exposure suppresses insulin secretion (Wei et al. 2017). Thus, the effect of insulin secretion by bisphenol A seems to involve a more complicated mechanism than previously thought.

Against this background, the present study was performed to delineate the mechanism underlying the induction and regulation of insulin gene transcription in insulinoma cells.

## Materials and Methods

### Reagents

Phenol red-free RPMI 1640 medium was obtained from Gibco (Grand Island, NY).

### Plasmid constructs

The human ERα expression plasmid, pHEGO, was kindly provided by Dr. P. Chambon (Green et al. 1986). Mutations in the P-box (C267S) and D-box (C202L) of the DBD of ERα were introduced using the QuikChange Site-Directed Mutagenesis Kit (Stratagene, La Jolla, CA) and the following primers: C267S: 5′-CCACCAACCAGTCGACCATTGATAAA-3′ and 5′-ATCGGATCCGCCAAGGAGACTCGCTAC-3′; C202L: 5′-GAGTCTGGTCCCTCGAGGGCTGCAA-3′ and 5′-ATCGGATCCGCCAAGGAGACTCGCTAC-3′.

To construct a mammalian expression vector for the Gal4 DBD fusion protein, the PCR-amplified LBD of ERα was inserted in-frame into the *Bam*HI and *Xba*I cloning sites of the pM vector (Clontech, Palo Alto, CA). Oligomers containing a *Bam*HI site and 18 base pairs (bp) of coding strand sequence of the N-terminus of the human ERα (hERα) hinge domain or an *Xba*I site and 18 bp of non-coding strand sequence of the hERα C-terminus were synthesized and used as PCR primers. Aliquots of each primer (100 pmol) were hybridized to 5 ng of ERα complementary DNA (cDNA) and amplified by PCR for 30 cycles (denaturation for 15 s at 95°C, annealing for 30 s at 55°C, and extension for 60 s at 72°C) using a PerkinElmer Gene Amp PCR System 2400 (PerkinElmer, Waltham, MA). After digestion with *Bam*HI and *Xba*I, the PCR products were ligated into the corresponding sites of the pM vector. Correct insertion was confirmed by dideoxy nucleotide sequencing. Mammalian expression vectors for peroxisome proliferator activated receptor alpha (PPARα), PPARγ, retinoid X receptor alpha (RXRα), retinoic acid receptor (RAR), glucocorticoid receptor (GR), and vitamin D3 receptor (VDR) were described previously (Miyamoto et al. 1997). PDX1 (Miller et al. 1994), BETA2 (Lee et al. 1995), and E47 (Voronova and Baltimore 1990) cDNAs were isolated from human pancreas mRNA by reverse transcription-polymerase chain reaction (RT-PCR). RT-PCR was performed as described previously (Miyamoto et al. 2001). The following primers were used to amplify the PDX1, BETA2, and E47 cDNAs: PDX1 forward primer 5′-TGAGGATCCATGAATAGTGAGGAGCAG-3′, PDX1 reverse primer 5′-TGTGTCGACTACCGGGGTTCCTGCGG-3′; BETA2 forward primer 5′-ATCGAATTCATGACCAAATCATACAGCG-3′, BETA2 reverse primer 5′-ATCGTCGACCTAATCGTGAAAGATGGC-3′; and E47 forward primer 5′-ATCGAATTCATGAACCAGCCGCAGAG-3′, E47 reverse primer 5′-ATCGTCGACTCACATGTGCCCGGC-3′.

### Deletion mutants

We generated insulin promoter-deletion mutants by PCR with *Xho*I-forward primer and *Hind*III-reverse primer. Genomic DNA was used as the template. Subsequently, we cloned the deletion mutants into the pGL3-basic Luciferase vector via the *Xho*I and *Hind*III sites and the following primers: *Hind*III transcriptional start site: ATCAAGCTTCTGGGGGTTACTGAATCC, XhoI-Ins 144: ATCCTCGAGGACCTAGCACCAGGCAAG, XhoI-Ins 188: ATCCTCGAGCTAAGTAGAGGTGTTG, XhoI-Ins 238: ATCCTCGAGGGTTCATCAGGCCACCCA, and XhoI-Ins 695: ATCCTCGAGGATCCCCCAACCACTCC.

### Cell culture

Hamster HIT-T15 insulinoma cells were obtained from the American Type Culture Collection (Manassas, VA; CRL1777) and cultured at 37°C in 5% CO_2_, 95% air in RPMI 1640 medium (11.1 mM glucose) supplemented with 10% charcoal-stripped fetal calf serum (Gibco), 100 units/ml penicillin, and 0.1 mg/ml streptomycin. HIT-T15 cells at passages 72–82 were used in the experiments. The rat INS-1 insulinoma cells were obtained from Dr. Hideo Mogami (Hamamatsu University School of Medicine). INS-1 cells were maintained in phenol red-free RPMI 1640 (11.1 mM glucose) supplemented with 100 units/ml penicillin–streptomycin and 10% dextran charcoal-stripped bovine calf serum. Cultures were maintained at 37°C and 7% CO_2_.

### RT-PCR experiments

RT-PCR mixtures were prepared as described previously (Miyamoto et al. 2001). The following forward and reverse primers were used for the PCR step: rat ERα sense primer 5′-ATCGGATCCGCCAAGGAGACTCGCTAC-3′, rat ERα antisense primer 5′-GTGCTTCAACATTCTCCCTCCTC-3′; rat ERβ sense primer 5′-GTCCTGCTGTGATGAACTAC-3′, rat ERβ antisense primer 5′-CCCTCTTTGCGTTTGGACTA-3′.

### Northern blot analysis

Total RNA was isolated using an RNeasy Kit (Qiagen, Valencia, CA). Fifteen-microgram aliquots of total RNA were size-fractionated in a 1% denaturing agarose-formaldehyde gel, transferred onto a Hybond-N+ nylon membrane (Amersham Pharmacia Biotech, Piscataway, NJ), and cross-linked with ultraviolet radiation (Stratalinker; Stratagene). Hybridizations were performed in ExpressHyb solution (Clontech) at 65°C for 2 h with full-length rat preproinsulin cDNA labeled with ^32^P-dCTP by random primer labeling (Amersham Pharmacia Biotech). After hybridization, membranes were washed at 65°C in 0.1× SSC buffer containing 0.1% SDS. The results were visualized using a Phosphor Imager (Fuji BAS 1500; Fuji, Tokyo, Japan). Northern blots were stripped and re-probed with GAPDH cDNA to control for RNA loading.

### Western blotting of nuclear extracts

Western blotting analysis was performed as described previously (Miyamoto et al. 1991) using a polyclonal antibody against human ERα (Santa Cruz).

### Preparation of nuclear extracts

Nuclear extracts were prepared as described previously (Sakuma et al. 2003).

### Transient-expression assay

HIT-T15 cells were transfected as described previously (Miyamoto et al. 2001), with minor modifications (Jiang et al. 2006).

### Luciferase and beta-galactosidase assays

Luciferase and beta-galactosidase assays were performed as described previously (Miyamoto et al. 2001).

### In vitro translation and GSTpull-down assays

In vitro translation and GST pull-down assays were performed as described previously (Kakizawa et al. 2001), with minor modifications (Jiang et al. 2006).

### Statistical analysis

Data are presented as the mean ± SD unless otherwise stated. Data were analyzed by Student’s *t* test. In all analyses, *P* < 0.05 was taken to indicate statistical significance.

Microsoft Excel 2013 and SPSS version 22.0 for Windows (IBM Japan, Tokyo, Japan) were used for the analyses. All *P* values shown in Fig. 3 were subjected to Bonferroni’s adjustment.

## Results

### Expression of ERα in HIT-T15 and INS-1 insulinoma cells and rat pancreatic islet cells

**Fig. 1:**
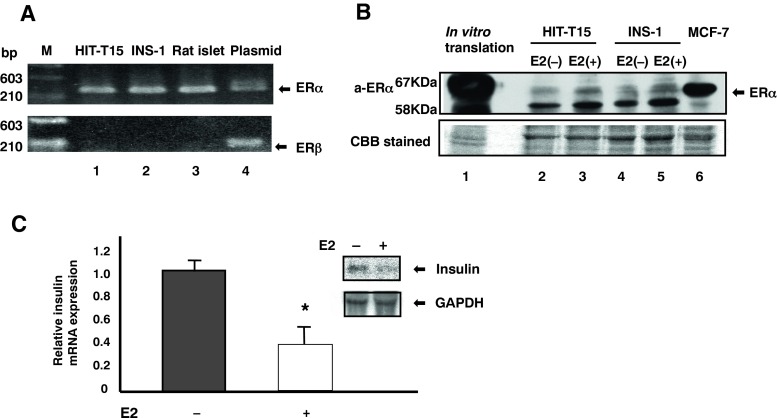
(*A*) RT-PCR analysis of ERα and ERβ mRNA expression in β cell lines. Total RNA from HIT-T15 cells (*lane 1*), INS-1 cells (*lane 2*), and rat islet cells (*lane 3*). PCR was performed using primers specific for ERα or preproinsulin cDNA, as described in the “Materials and Methods.” Plasmids containing ERα or ERβ cDNA were used as templates for positive control reactions. (*B*) Western blotting analysis of ERα protein expression in pancreatic β cell lines. Nuclear extracts from HIT-T15 (*lanes 2* and *3*) and INS-1 cells (lanes 4 and 5) were analyzed using a polyclonal antibody against human ERα. The position of ERα is indicated. Specificity of the antibody was evaluated using 5 ml of in vitro translated ERα (*lane 1*) expressed with am unprogrammed reticulocyte lysate. (*C*) Effects of an ER agonist on insulin mRNA levels. Total RNA (15 mg per *lane*) was isolated from control cells (*lane 1*) or cells treated with or without 10^−7^ M E2. Isolated RNA was separated by electrophoresis, blotted onto nylon membranes, and hybridized with ^32^P-labeled preproinsulin cDNA. β-Actin levels were detected to ensure equal loading of RNA in each lane. *P < 0.05 and ** *P* < 0.01, based on Student’s *t* test.

We first examined the expression of ERα and ERβ in clonal HIT-T15 pancreatic β islet cells. As shown in Fig. 1*A*, RT-PCR analysis indicated that ERα mRNA was abundantly expressed in HIT-T15, INS-1 cells, and rat pancreatic β islet cells, whereas little if any ERβ mRNA expression was observed. The predicted sizes of the PCR products for ERα and ERβ were 273 and 285 bp, respectively. Furthermore, western blotting analysis demonstrated the presence of the ERα protein in the nuclear extracts of HIT-T15 and INS-1 cells (Fig. 1*B*). ER mRNA- and protein-expression levels in both HIT-T15 insulinoma cells and normal β islet cells were comparable to those seen in MCF-7 breast cancer cells (data not shown). The specificity of the antibody was verified using a recombinant ERα protein translated in vitro using an unprogrammed reticulocyte lysate.

### Effects and localization of E2 on insulin expression in insulinoma cells

We hypothesized that nuclear ER signaling may be involved in regulating insulin production in HIT-T15 cells. Firstly, to analyze the effects of E2 (an ER agonist) on insulin mRNA expression, we performed northern blotting analysis of total RNA from HIT-T15 insulinoma cells incubated for 48 h with E2. As shown in Fig. 1*C*, the expression of preproinsulin mRNA in HIT-T15 cells decreased significantly following treatment with 10^−7^ M E2.

### ER repressed transcriptional activity of the rat insulin II promoter in a ligand-dependent manner in transient-expression assays in HIT-T15 cells

**Fig. 2:**
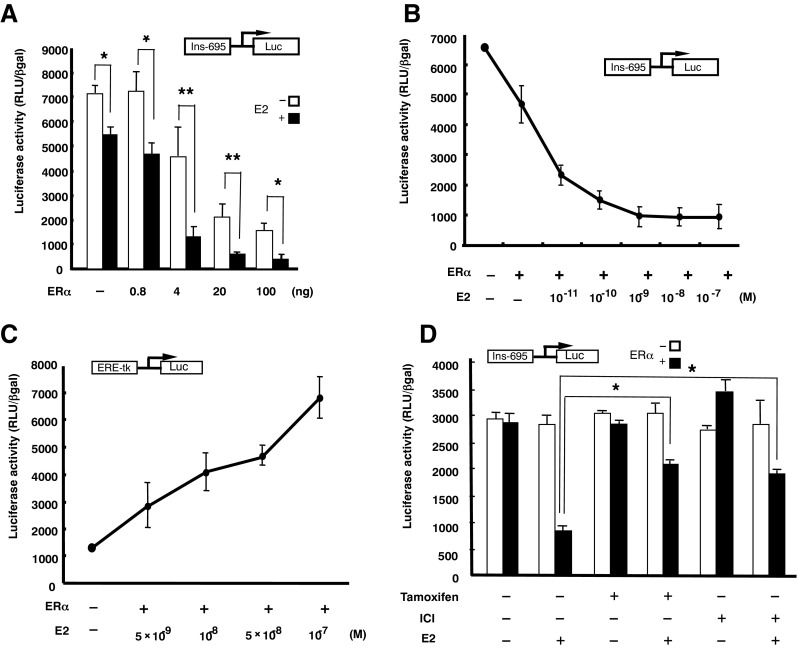
(*A*) ERα repressed transcriptional activity of the insulin promoter in a ligand-dependent manner. HIT-T15 cells were transfected with 0.25 mg of a luciferase reporter plasmid under the transcriptional control of the rat insulin gene promoter (–695INS-Luc) along with an ERα expression plasmid. Twelve hours later, the cells were treated with vehicle control or 10^−7^ M E2 and collected for analysis of reporter gene activity 24 h later. The total amount of transfected DNA was held constant by adding empty expression vector to the transection cocktail where needed. Triplicate wells were transfected, and the data are expressed as the mean ± SD of at least four individual experiments. (*B*) E2 repressed transcriptional activity of the insulin promoter in a dose-dependent manner. HIT-T15 cells were cotransfected with 0.25 mg of the luciferase reporter plasmid, –695INS-Luc, and 4 ng of the ERα expression plasmid. Twelve hours later, the cells were treated with vehicle or 10^−11^–10^−7^ M E2 and collected for analysis of reporter gene activity 24 h later. The total amount of transfected DNA was held constant by adding empty expression vectors to the transfection mixture. Triplicate wells were transfected, and the data are expressed as the mean ± SD of at least four independent experiments. (*C*) COS 1 cells, an African green monkey kidney fibroblast-like cell line, were transfected with 0.25 mg of luciferase reporter plasmid (ERE-TK-Luc) and 100 ng of the ERα expression plasmid. Cells were treated with the indicated concentrations of E2 and collected for reporter gene assays 24 h later. Triplicate wells were transfected, and the data are expressed as the mean ± SD of at least four individual experiments. (*D*) ERα repressed transcriptional activation of the rat insulin II promoter with E2, whereas 10^−6^ M tamoxifen and 10^−7^ M ICI 182,780 inhibited the effect of E2. After a 12-h transfection period, the cells were treated with vehicle or 10^−7^ M E2, 10^−6^ M tamoxifen, or 10^−7^ M ICI 182,780 and collected for reporter gene assays 24 h later. Triplicate wells were transfected, and the data are expressed as the mean ± SD of at least four individual experiments. *P < 0.05 and **P < 0.01, based on Student’s *t* test.

Transient-expression experiments were performed in HIT-T15 cells using luciferase reporter plasmids containing the promoter region of the rat insulin II gene, extending from − 695 to + 1 bp relative to the transcriptional start site. Forced overexpression of ERα repressed the promoter further: the repression was dose-dependent with the maximum suppression being 20% with 100 ng ERα (Fig. 2*A*). As shown in Fig. 2*A*, the addition of 10^−7^ M E2 suppressed activity of the transfected reporter gene by approximately 20% without transfection of the ERα-expression vector, pHEGO (*P* < 0.05). This repression was likely mediated by endogenous ERα. The repression caused by overexpression of ERα was further suppressed by the presence of 10^−7^ M E2. In contrast, E2 did not alter the T3-dependent transcriptional activity in controls (data not shown), suggesting that the repression was specific for the insulin promoter and was not due to a toxic effect of E2 in the cells. Cell viability was unaffected by the addition of E2.

### ER repressed insulin promoter activities in either an E2-dependent or E2-independent manner

E2 exposure in the range of 10^−11^–10^−7^ M decreased transcription driven by the − 695 to + 1 promoter region by up to 80%. ER-transfected HIT-T15 cells not treated with estrogen showed partial reduction of insulin promoter activity to a level approximately 70% of that in control cells (Fig. 2*B*). These results suggest that (1) estrogen reduced insulin promoter transcription in an ER-dependent manner and (2) transcriptional suppression by ER occurred even in the absence of estrogen.

### Tamoxifen and ICI 182,780 inhibited the effect of E2

Next, we compared the titration curves for other receptors and ligand in terms of inhibiting the insulin promoter, relative to the activation of the luciferase reporter plasmid (ERE-TK-Luc) by ERα. The concentration range over which E2 inhibited the insulin promoter and that required for ERE activation was comparable (Fig. 2*C*).

To check whether the inhibitory effect of E2 acted at AP1 sites, we performed transient-expression assays with tamoxifen and ICI 182,780. As shown in Fig. 2*D*, ERα repressed transcriptional activation of the rat insulin II promoter by E2, while tamoxifen and ICI 182,780 inhibited the effect of E2. To test the specificity of the inhibitory effect among nuclear receptors, we examined the abilities of RXRα, VDR, RAR, and GR to repress insulin gene promoter activity in the absence or presence of their cognate ligands. As shown in Fig. S1, none of the nuclear receptors tested showed inhibition of the rat insulin II promoter.

### ERα did not affect the transcription of other NRs

The TK promoter containing the thyroid hormone-response element (TRE), glucocorticoid-response element (GRE), or peroxisome proliferator-response element (PPRE) was evaluated to determine whether transcriptional repression is a general phenomenon induced by E2/ERα in HIT-T15 cells. The transcriptional activities of the TRE-, GRE-, and PPRE-TK promoters were unaffected by E2 treatment (Fig. S2), excluding such a possibility.

### Localization of the insulin 5′ promoter region involved in estrogen repression

**Fig. 3:**
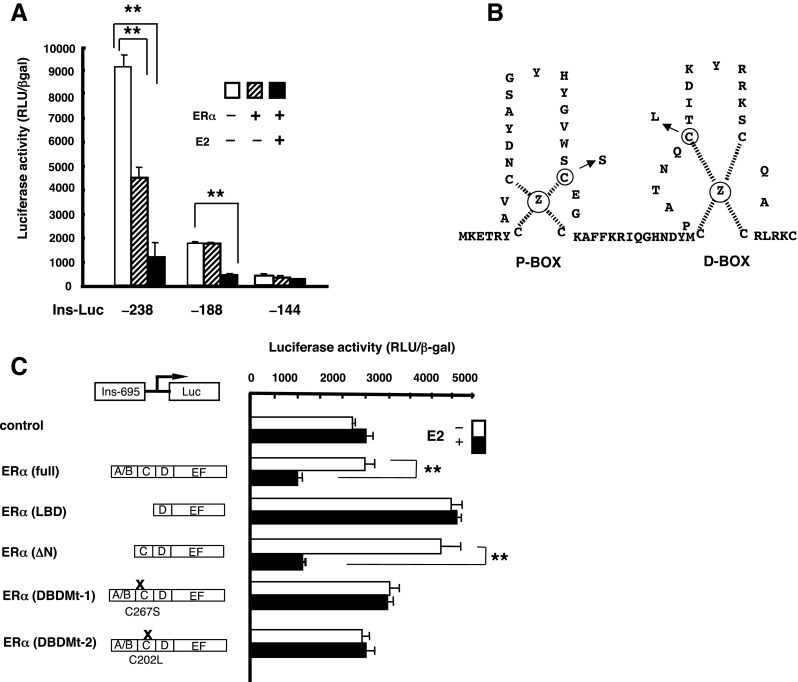
(*A*) Deletion analysis of insulin gene activity in HIT-T15 cells. HIT-T15 cells were cotransfected with a series of 5′ deletion mutants of the insulin promoter luciferase reporter and the ERα expression vector. Cells were treated with 10^−7^ M E2 for 24 h, and then luciferase activities were measured. Triplicate wells were transfected and the data are expressed as the mean ± SD of at least three individual experiments (*P < 0.05 and **P < 0.01; all *P* values were subjected to Bonferroni’s adjustment). (*B*) ERα DBDMtC227S was engineered by changing cysteine at position 227 of the DNA-binding helix of ERα to serine, whereas ERα DBDMtC202L contained a replacement of cysteine at position 202 with leucine. (*C*) Mutation of the DNA-binding region in ERα abolished the repression. HIT-T15 cells were cotransfected with the empty expression vector, ERα expression vector, or the indicated mutant ER expression vector. Cells were treated with 10^−7^ M E2 for 24 h, after which luciferase activities were measured. Triplicate wells were transfected, and the data are expressed as the mean ± SD of at least four individual experiments (*P* < 0.01). *P < 0.05 and ** *P* < 0.01, based on Student’s *t* test.

To identify the insulin promoter region mediating estrogen-dependent downregulation of insulin gene transcription in HIT-T15 cells, progressive 5′ promoter deletion constructs were cotransfected with the pHEGO vector (Green et al. 1986). Transcription driven by the − 238 insulin promoter decreased by nearly 20% at 10^−7^ E2 (Fig. 3*A*). Deletion of nucleotides − 695 to − 188 muted the repression by E2 (30% decrease). E2 did not significantly repress the activities of the insulin promoter variants containing additional progressive 5′ deletions (to − 144). The data indicated that the estrogen-responsive region of the insulin promoter was located between nucleotides − 238 and − 144, that nucleotides − 238 to − 188 contained sites for E2-dependent and independent repression, and that nucleotides − 188 to − 144 contained an E2-dependent repression site.

### An intact ER DBD was essential for the repression of insulin gene transcription

To evaluate the requirement of the DBD in repression of the insulin promoter by E2, cotransfection experiments were conducted using vectors encoding an N-terminal truncation mutant of ER (ΔN ERα). In the presence of ΔN ER, E2 repressed insulin promoter activity by up to 80%, similar to that shown by the wild-type ER (Fig. 3*C*), suggesting that the N-terminus (A/B domain) of ER was not required for repression. These data indicate that an intact DBD of ER was required for E2-dependent repression of insulin transcription (Fig. 3*C*). To further localize the domains required for the inhibition of insulin gene transcription by ERα, mutated receptors were generated (Fig. 3*B*). The repression was lost upon introducing mutations in the P-box of the first zinc finger and the D-box of the second zinc finger in the DBD (Fig. 3*C*). These data indicate that the DBD (C domain) was necessary for the repression.

**Fig. 4:**
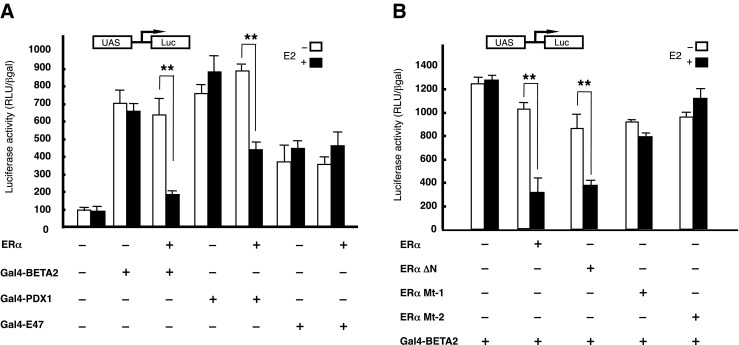
(*A*) COS7 cells were transfected with 100 ng of vector encoding the TK-UAS-Luc gene, GaL4-BETA2, GaL4-PDX1, or GaL4-E47 or 100 ng/well of the pHEGO expression plasmid. Cells were treated with or without 10^−7^ M E2 for 24 h. The data shown represent the mean ± SD from two independent studies, performed with triplicate samples. (*B*) COS7 cells were transfected with 100 ng/well of the TK-UAS-Luc gene, GaL4-BETA2, and pHEGO expression plasmids or the indicated mutant ER expression vectors. Cells were treated with or without 10^−7^ M E2 for 24 h. The data shown represent the mean ± SD from two independent studies, performed with triplicate samples.

Next, we examined whether ER alters the activity of transcription factors such as PDX1 and E47/BETA2, which bind to the repressive region and its vicinity on the insulin promoter (Fig. S3). Plasmids for expressing Gal4 DBD fusions of these transcription factors were generated and cotransfected with the ERα expression vector and the UAS reporter plasmid. As shown in Fig. 4*A*, ERα repressed the activities of PDX1 and BETA2 in an E2-dependent manner. Moreover, the repression was lost upon mutation of the first and second zinc finger domains of the ERα DBD (Fig. 4*B*). These observations raised the possibility that ERα interacts with PDX1 and BETA2.

**Fig. 5:**
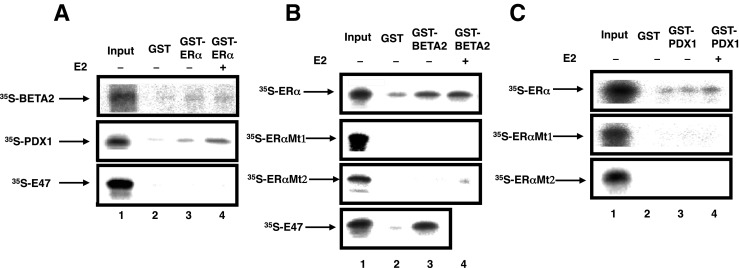
(*A*) BETA2 and PDX1 interacted directly with ERα in vitro. ^35^S-labeled BETA2 or PDX1 was incubated with matrix-bound GST-ERα with E2 (*lane 4*) or without E2 (*lane 3*), and 10% of the input ^35^S-labeled proteins is indicated (*lane 1*). Associated proteins were analyzed by 10% sodium dodecyl sulfide-polyacrylamide gel electrophoresis (SDS-PAGE) and visualized with a BAS 1500 (Fuji). (*B*) ^35^S-labeled ERα or ERαMt was incubated with matrix-bound GST-BETA2 with (*lane 3*) or without E2 (*lane 4*) 10% of the input ^35^S-labeled proteins, as indicated (*lane 1*). As a control, ^35^S-labeled E47 interacted with GST-BETA2. Associated proteins were analyzed by 10% SDS-PAGE and visualized using a BAS 1500 (Fuji). (*C*) ^35^S-labeled ERα or ERαMt was incubated with matrix-bound GST-PDX1 with (*lane 3*) or without E2 (*lane 4*), and loading with 10% of input ^35^S-labeled proteins is indicated (*lane 1*). Associated proteins were analyzed by 10% SDS-PAGE and visualized using a BAS 1500 (Fuji).

As the results of the transient-expression assays suggested that interactions between ERα and PDX1 or BETA2 occur, we performed GST pull-down assays to examine whether ERα could directly interact with PDX1 or BETA2. The matrix-bound fusion protein between glutathione *S*-transferase and ERα (GST-ERα) was used for in vitro pull-down assays. As shown in Fig. 5*A*, ^35^S-methionine-labeled, in vitro-translated BETA2 and PDX1 interacted with GST-ERα in the presence of E2. In addition, ^35^S-methionine-labeled ERα interacted with GST-BETA2 (Fig. 5*B*) and GST-PDX1 (Fig. 5*C*). These data indicated that direct protein–protein interactions occurred between ERα and PDX1 or BETA2.

## Discussion

Recent findings have shown that estrogen is important for pancreatic β cells in mammals (Nadal et al. 2009). Long-term exposure to physiological concentrations of E2 increased β cell insulin contents, insulin gene expression, and insulin release (Alonso-Magdalena et al. 2008). E2 protected pancreatic β cells from apoptosis and prevented insulin-deficient diabetes mellitus in mice (Le May et al. 2006). GPER1-deficient mice lost E2-stimulated insulin release, suggesting that GPER1 mediates the E2 response in pancreatic islets (Martensson et al. 2013). Wong et al. reported that islet ERα enhances insulin biosynthesis in vitro, and it amplifies the stimulatory effect of glucose on the insulin gene promoter (Wong et al. 2010). Kilic et al. reported that islet ERα was induced by hyperglycemia and protected against oxidative stress-induced insulin-deficient diabetes (Kilic et al. 2014). However, data from some studies suggested that E2 negatively affected glucose-stimulated insulin secretion. Short-term supraphysiological estrogen administration can adversely affect glucose tolerance, resulting from the suppression of first-phase insulin secretion and increased insulin resistance (Godsland 2005). Resveratrol inhibited insulin secretion from rat pancreatic islets (Szkudelski 2007). Exposure to bisphenol A (BPA) induced dysfunction of insulin secretion and apoptosis by damaging mitochondria in rat insulinoma (INS-1) cells (Lin et al. 2013). Long-term oral exposure to BPA induced glucose intolerance and insulin resistance (Moon et al. 2015). Recently, Wei et al. reported that short-term BPA exposure downregulated miR-338 through upregulation of the G protein-coupled estrogen receptor 1, PDX1, causing increased insulin secretion. In contrast, long-term BPA exposure upregulated miR-338 through suppression of the glucagon-like peptide 1 receptor and PDX1, resulting in suppressed insulin secretion (Wei et al. 2017).

### Estrogen treatment led to reduced insulin mRNA levels in HIT-T15 cells

In this study, we critically reevaluated the effects of estrogen through ERα on insulin gene transcription and demonstrated that estrogen treatment led to reduced insulin mRNA levels in HIT-T15 cells. Suppression of insulin gene transcription by ERα was specific among the nuclear receptors tested and, therefore, seemed to be independent of the interactions with common nuclear receptor mediators.

Many findings have indicated the indirect association of ER with DNA through other DNA-bound transcription factors. Estrogen has been reported to regulate the expression of genes harboring AP-1-binding elements, e.g., human collagenase, IGF-I, cyclin D1, matrix metalloproteinase-1, and choline acetyl-transferase genes, the chicken ovalbumin gene, and the bovine FSH β gene. In addition, ER enhanced the transcription of genes containing SP1-binding sites (Bjornstorm and Sjoberg 2005).

Tamoxifen is a potent activator of estrogen receptor (ER)-mediated induction of promoters regulated by AP-1 sites (Barsalou et al. 1998). ICI 182,780 is an antagonist of both ERα and ERβ when the receptors are tethered to the AP-1 (Jakacka et al. 2001), Sp1 (Hay and Docherty 2006), and STAT5 (Peshavaria et al. 1994) transcription factors in the nucleus. In our transient-expression experiments, ER antagonists, such as tamoxifen and ICI 182,780, inhibited the effect of E2, suggesting that the mechanism of insulin promoter repression is different from that of suppressing promoters containing AP-1, Sp1, and STAT5.

We found that a construct containing only the D, E, and F domains of ER did not affect transcription from the insulin promoter reporter despite hormonal treatment, whereas the construct lacking the A/B domain (but containing the DBD) altered transcription in the same manner as the wild-type ER (Fig. 3*C*). These findings were consistent with those from several previous reports (Caldenhoven et al. 1995; Scheinman et al. 1995; Faulds et al. 2001; Gonzalez and Carlberg 2002) and raise the possibility that interactions of the ER with these factors involve a region within the DBD. It is also notable that the C227S and C202L mutations eliminated activity through the non-classical pathway. With the suppressive effect of E2 on insulin promoter activity, the zinc fingers may participate in protein–protein interactions.

We also demonstrated that the ER repressed insulin promoter activity in a ligand-dependent manner through a mechanism involving protein–protein interactions. We identified a region within the gene promoter that mediates transcriptional repression of insulin gene expression in an ER- and E2-dependent manner in HIT-T15 cells. By transiently expressing a series of deletion promoter–reporter constructs, we localized the optimal repressive activity between nucleotides − 238 to − 144 region in the promoter. Moreover, we demonstrated that pancreatic β cell-specific transcription factors (PDX1 and BETA2/E47), which interact with this element and its vicinity and regulate insulin gene transcription, are involved in the repression by ER.

The region of E2 responsiveness in the insulin promoter was localized to nucleotides − 238 to − 144 by deletion analysis. Within this region, there is a PDX1-binding site, A3 (nucleotides − 206 to − 197), that was shown previously to regulate basal insulin transcription (Peers et al. 1994).

We found that deletion of the PDX1 site between nucleotide positions − 238 to − 188 in the insulin promoter resulted in partial loss of E2-independent repression, comparable to the loss of repression observed after deletion of a broad region surrounding the site (nucleotides − 238 to − 144). Deletion of the region from − 695 to − 238 had no effect on E2-dependent repression. Therefore, the segment from − 238 to − 144 appears to play a role in E2-dependent repression of the insulin promoter.

Basal expression of the insulin gene is under the control of multiple transcription factors acting at multiple *cis*-acting elements (Crowe and Tsai 1989). Each of the regulatory elements appears to serve a minor role in transcriptional regulation, rather than any single element being responsible for a major role. This is consistent with our observation that deletions resulted in partial loss of repression, but the response was completely lost only after deletion of a broad region of the promoter.

There are parallels with ER-mediated repression of IL-6 that may help explain the mechanism of insulin repression. In both cases, the repression was lost and the promoters were actually activated in the presence of the DBD mutant of ER. The intact DBD is required for interaction of the ER with transactivators of the IL-6 gene (Galien et al. 1996), as well as for interaction of the ER with the transcription factor, STAT5, which regulates the expression of milk protein genes (Peers et al. 1994). A mutation in the ER that alters its interaction with other proteins can transform the receptor from a transcriptional activator to a repressor (Paech et al. 1997). Thus, mutation of the DBD may have transformed the ER from a repressor to an activator of the insulin promoter by altering its interaction with coactivators and/or corepressors. The ER may also repress insulin transcription by such a mechanism, perhaps involving interactions with the PDX1 and BETA2/E47 proteins, based on our observation that the repression is mediated by transcription factors. Possible candidates for the protein–protein interaction involved in repressing insulin gene expression include PDX1, E47, and BETA2, which bind to the vicinity of region from positions − 239 to − 144 bp in the insulin promoter. This is supported by evidence of ER repression with Gal4-PDX1, Gal4-E47, and Gal4-BETA2 in HIT-T15 cells. Attempts to supershift AP1-bound Jun with ER in electrophoretic mobility shift assays or to coimmunoprecipitate a PDX1–E47–BETA2-ER complex did not reveal direct interactions (data not shown). It is possible that these interactions were not strong enough to withstand the experimental conditions.

Wong et al. analyzed the effect of the ER on the insulin promoter using pancreas-specific ERα-knockout mice, cultured islets, and INS-1 insulinoma cells (Wong et al. 2010). Although our results suggest that BETA2 is closely related to ER signals in pancreatic β cells, ER and E2 repress insulin promoter activity. We could not reproduce the insulin promoter activation by transient expression of the ER in INS-1 cells because the luciferase reporter activities were very low.

Several reasons may explain the differences between our current results and previous findings. First, we think that because the glucose concentration of 11 mM used in our experimental system is higher than that found at physiological concentrations, insulin synthesis may have been considerably accelerated. E2 and ER activities under this condition may be inhibitory. Even the report by Wong et al. (2010) showed that 11 mM glucose did not strongly affect insulin promoter activity after transient ER expression in INS-1 cells. A second explanation of the differences found between the current and previous findings may relate to the duplication time of INS-1 and HIT-T15 cells. The INS-1 tumor cell line showed increased insulin secretion, but the HIT-T15 cell line displayed greater uptake of E2 and ER after transient ER expression, with faster turnover. Primary cultures of islet and INS-1 cells are closer approximations of cells found under normal physiological conditions, and HIT-T15 cells better reflect a state of insulin hypersecretion, such as insulinoma. Wei et al. (2017) reported that a 48-h exposure of pancreatic cells to BPA suppressed insulin secretion. PDX was involved in the suppression mechanism, which supports our experimental results regarding suppressing the insulin promoter via PDX1.

## Conclusion

In this study, we demonstrated that the expression of preproinsulin mRNA in insulinoma cells decreased significantly following treatment with E2 and that estrogen-based repression of rat insulin II promoter activity was mediated by a broad promoter region, which contains PDX1- and BETA/E47-binding sites. The ER DBD likely interacted with these proteins, and this interaction requires a structurally intact DBD. ER may regulate insulin transcription by indirect genomic signaling involving interactions with PDX1 and BETA2/E47 proteins. Collectively, our data indicate that estrogen indirectly repressed rat insulin gene transcription through the ER in HIT-T15 cells.

## Electronic supplementary material

ESM 1. (PDF 78 kb)
