# Supplementary material for: Repression of insulin gene transcription by indirect genomic signaling via the estrogen receptor in pancreatic beta cells
Source: In Vitro Cell Dev Biol Anim. 2019 Feb 21;55(4):226–36. doi: 10.1007/s11626-019-00328-5 (PMC6443913; doi:10.1007/s11626-019-00328-5)
Supplement: Supplementary file 1 — (PDF 78 kb) [file 11626_2019_328_MOESM1_ESM.pdf]

# Supplementary Figure 1

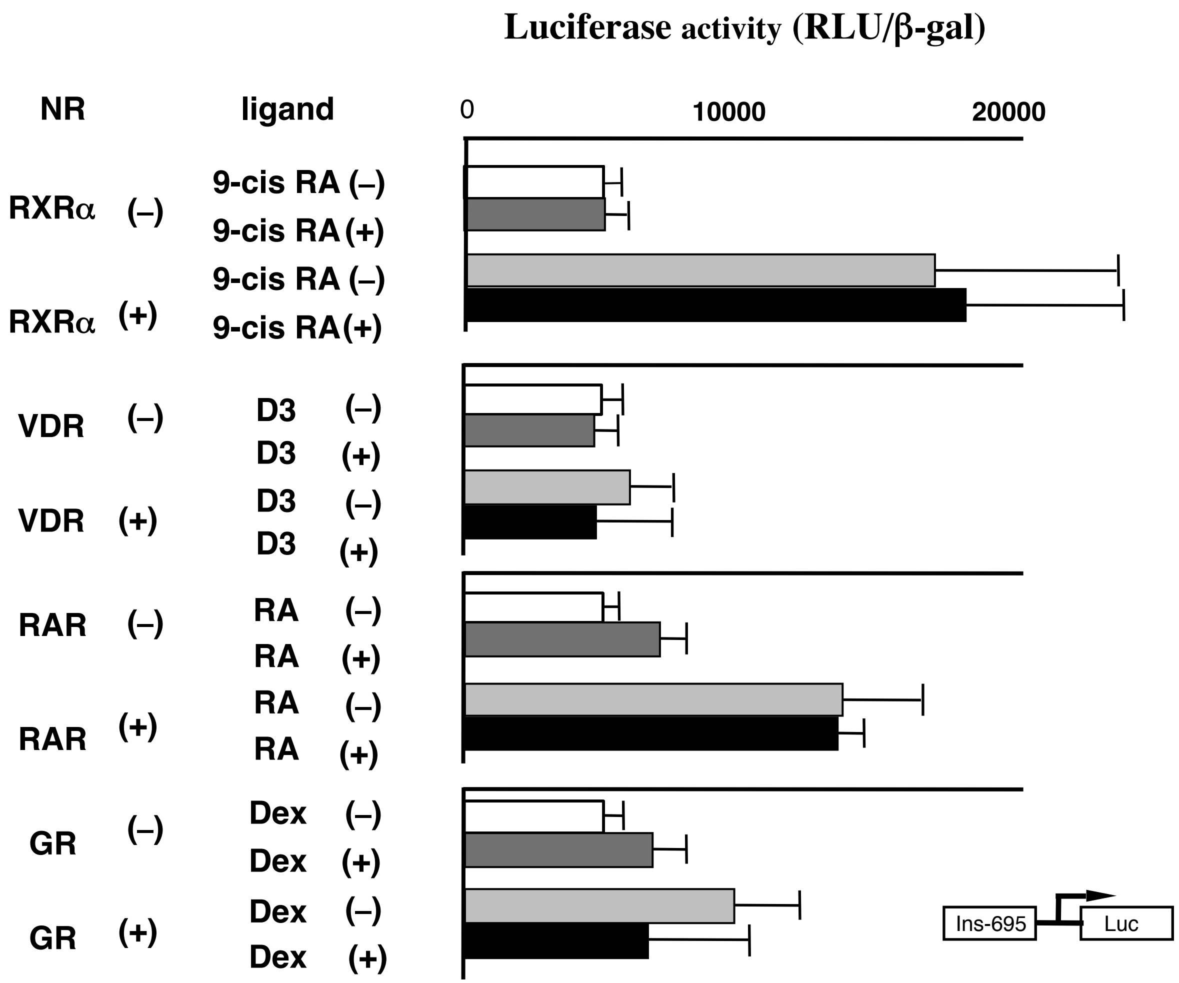


Sekido and Nishio *et al.*

# Supplementary Figure 2

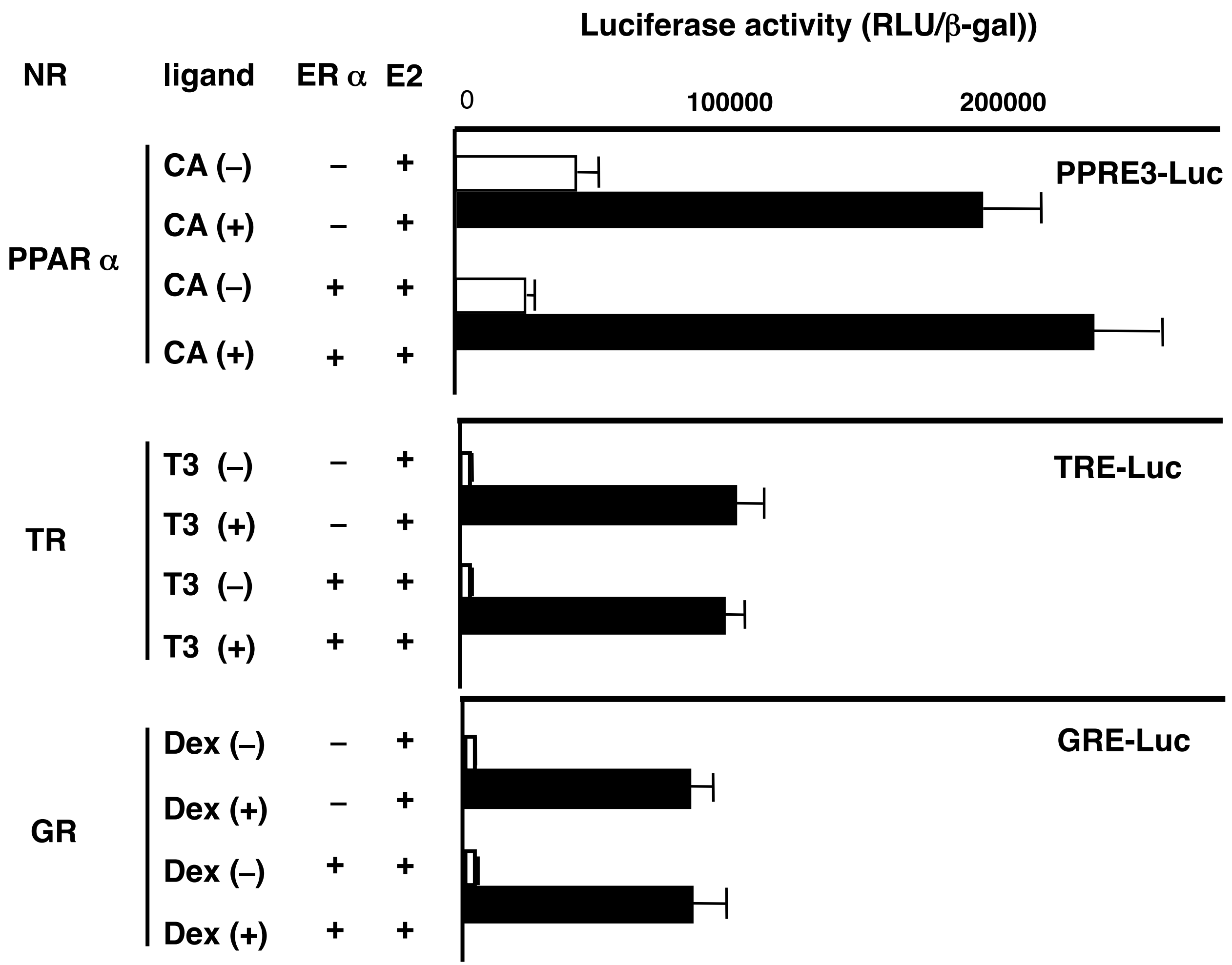


Sekido and Nishio *et al.*

# Supplementary Figure 3

## rat insulin II gene promoter

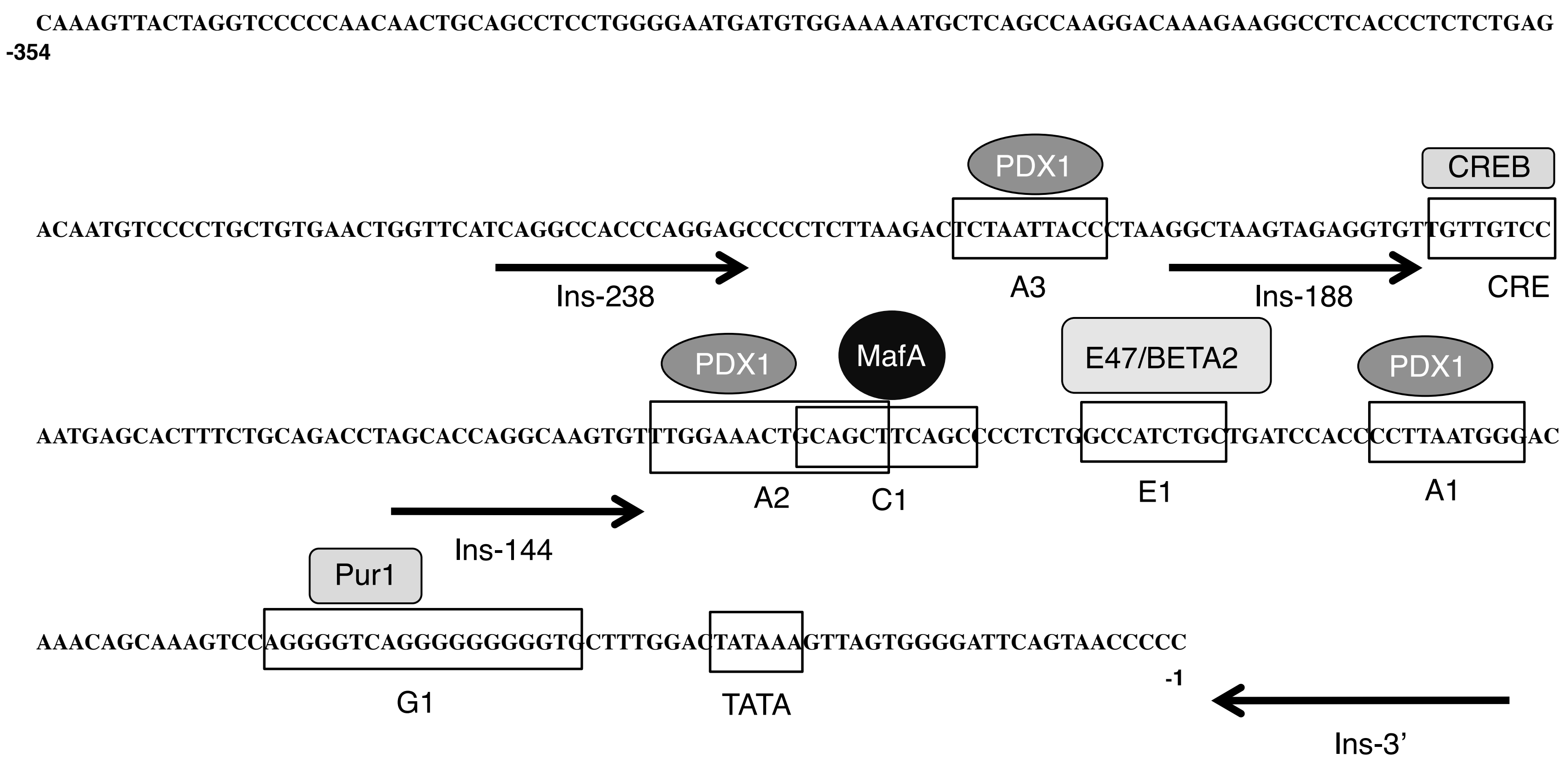


Sekido and Nishio *et al.*
